# Correlation of Triiodothyronine Level with In-Hospital Cardiac Function and Long-Term Prognosis in Patients with Acute Myocardial Infarction

**DOI:** 10.1155/2018/5236267

**Published:** 2018-12-02

**Authors:** Jianqing She, Jiahao Feng, Yangyang Deng, Lizhe Sun, Yue Wu, Manyun Guo, Xiao Liang, Jingjin Li, Yulong Xia, Zuyi Yuan

**Affiliations:** ^1^Cardiovascular Department, First Affiliated Hospital of Medical College, Xi'an Jiaotong University, Xi'an 710048, China; ^2^Key Laboratory of Environment and Genes Related to Diseases, Ministry of Education, Xi'an 710048, China; ^3^Cardiovascular Department, First Affiliated Hospital of Peking University, Beijing 100005, China

## Abstract

**Objective:**

The pathophysiologic mechanism of how thyroid function is related to the development and prognosis of acute myocardial infarction (AMI) remains under explored, and there has been a lack of clinical investigations. In this study, we investigate the relationship between triiodothyronine (T3) level and cardiac ejection fraction (EF) as well as probrain natriuretic peptide (NT-proBNP) on admission and subsequent prognosis in AMI patients.

**Methods:**

We measured admission thyroid function, NT-proBNP, and EF by echocardiography in 345 patients diagnosed with AMI. Simple and multiregression analyses were performed to investigate the correlation between T3 level and EF as well as NT-proBNP. Major adverse cardiovascular events (MACE), including new-onset myocardial infarction, acute heart failure, and cardiac death, were documented during the follow-up. 248 participants were separated into three groups based on T3 and free triiodothyronine (FT3) levels for survival analysis during a 2-year follow-up.

**Results:**

345 patients diagnosed with AMI were included in the initial observational analysis. 248 AMI patients were included in the follow-up survival analysis. The T3 levels were found to be significantly positively correlated with EF (*R* square = 0.042, *P* < 0.001) and negatively correlated with admission NT-proBNP levels (*R* square = 0.059, *P* < 0.001), which is the same with the correlation between FT3 and EF (*R* square = 0.053, *P* < 0.001) and admission NT-proBNP levels (*R* square = 0.108, *P* < 0.001). Kaplan-Meier survival analysis revealed no significant difference with regard to different T3 or FT3 levels at the end of follow-up.

**Conclusions:**

T3 and FT3 levels are moderately positively correlated with cardiac function on admission in AMI patients but did not predict a long-time survival rate. Further studies are needed to explain whether longer-term follow-up would further identify the prognosis effect of T3 on MACE and all-cause mortality.

## 1. Introduction

Acute myocardial infarction (AMI) is one of the leading health-threatening diseases worldwide [1, 2]. Although it has been well known that atherosclerosis contributes to the pathogenesis of AMI [3, 4], metabolism disorders including deregulated thyroid function have also been reported to play direct and indirect effects in the heart and vasculature lesions [5–7]. However, the pathophysiologic mechanism of how thyroid function is related to the development and prognosis of myocardial infarction remains under explored, and there has been a lack of clinical investigations.

Biologically active thyroid hormones include thyroxine (T4) and triiodothyronine (T3) [8, 9]. It has been reported that thyroid hormone activity in the cardiomyocyte regulates myocardial contractility, diastolic, and systolic function. Moreover, thyroid hormones also exert profound effects on the heart and on cardiovascular hemodynamics, maintaining cardiac mass and wall stress while preserving ellipsoid left ventricular geometry [10, 11].

While T4 is solely a product of the thyroid gland, T3 is a product of the thyroid and of many other tissues. Low T3 level has been proven to be correlated with myocardial damage in animal models [12] and is associated with a poor cardiovascular prognosis in human [11, 13, 14]. Previous studies have indicated that subclinical hypothyroidism could be a predictor of adverse cardiovascular outcomes in patients with acute decompensated heart failure [14–16], and low T3 has been correlated with worse hospital outcomes in patients with acute HF [11, 14]. However, most of the studies focused on the function of T3 in patients with heart failure, while there has been a lack of evidence with regard to patients with acute myocardial infarction.

In this prospective cohort study, we investigated the relationship between triiodothyronine (T3) level and cardiac ejection fraction (EF) as well as N-terminal fragment of probrain natriuretic peptide (NT-proBNP) on admission. We then carried out survival analysis to investigate the prognostic value of T3 and FT3 level on long-term mortality and morbidity in AMI patients.

## 2. Research Design and Methods

### 2.1. Study Design and Participants

This was a single-centre prospective cohort study. Consecutive patients admitted to the cardiology department of the First Affiliated Hospital of Xi'an Jiaotong University for AMI between January 2013 and December 2016 were selected. The inclusion criteria and exclusion criteria were as previously described [17]. AMI were defined based on the universal definition criteria by the American Cardiology College [18].

### 2.2. Assessment of Thyroid Function, Cardiac EF, and NT-proBNP

Blood thyroid function of all patients was measured by Immulite 2000 (Bio DPC, Los Angeles, USA) within 24 h of admission after overnight fasting. The reference intervals of our laboratory were as follows: T3 0.78–2.20 ng/mL, T4 4.2–13.5 *μ*g/dL, FT3 2.91–9.08 pmol/L, FT4 9.05–25.5 pmol/L, TSH 0.25–5 mIU/mL, TGAB < 30%, and TMAB < 20%. The patients were further divided into 3 groups, respectively, according to tertiles of T3 and FT3. NT-proBNP levels of all patients were measured using immunoassay (Elecsys® ProBNP, Roche Diagnostics, Indianapolis, IN, USA) upon initial admission without fasting, and the reference interval was 0–125 pg/mL. Because NT-proBNP values were not normally distributed, NT-proBNP levels were log-transformed to the base 2, and analyses were reported per doubling of NT-proBNP. Echocardiographs were performed on admission by experienced echo cardiologists, and systolic function was expressed as the ejection fraction (EF), which was calculated using Simpson's method.

### 2.3. Statistical Analysis

All statistical analyses were performed by using SPSS 17.0 for Windows (SPSS Inc., Chicago, IL). Data were presented as frequencies and percentages for categorical variables and mean ± SD for continuous variables unless otherwise indicated. One-way ANOVA was used to compare continuous variables. Simple linear analysis was used for calculating correlation between T3 level and EF, T3 level and NT-proBNP, FT3 level and EF, and FT3 level and NT-proBNP, respectively. To ascertain the independent contribution to EF and NT-proBNP, multivariate regression analysis was conducted. Kaplan-Meier survival curve analysis was used to represent the proportional risk of all-cause mortality and MACE for the admission T3 and FT3 values in AMI patients. Patients were divided into three groups based on tertiles of T3 and FT3 levels. A value of *P* < 0.05 was considered statistically significant.

## 3. Results

### 3.1. Study Population

**Figure 1:**
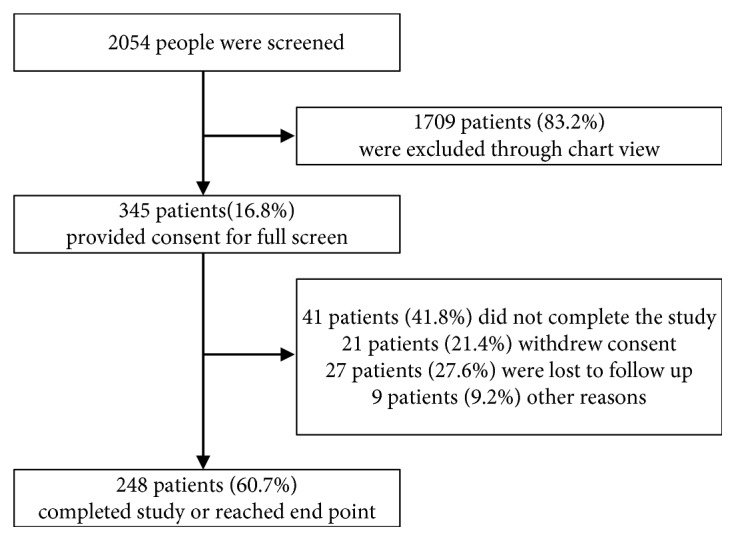
Enrolment and outcomes.

From January 2013 till December 2016, a total of 2054 patients were enrolled in the study; 345 patients, including 139 STEMI and 206 non-STEMI patients, provided consent for full screen and were included in the initial observational analysis, while 248 patients were included in the follow-up survival analysis (Figure 1). All of the patients have received coronary angiography, and 95.36% patients had coronary intervention. Patients were divided into three groups based on tertiles of T3 and FT3 levels for the survival analysis. Baseline patients' characteristics are shown in Table 1 based on the T3 levels (0.500–0.860 ng/mL, 0.862–1.140 ng/mL, and 1.150–2.190 ng/mL) and in Table 2 based on the FT3 levels (1.62–3.98 pmol/L, 3.99–5.33 pmol/L, and 5.34–8.55 pmol/L). The mean age was 58.766 ± 10.313, 59.477 ± 10.039, and 59.725 ± 10.237 years in respective T3 groups and was 59.625 ± 10.468, 58.351 ± 10.107, and 59.694 ± 10.490 in FT3 groups. No significant difference in risk factors at baseline was seen in different T3 and FT3 groups except for aspartate transaminase (AST), EF, NT-proBNP, and thyroid hormones.

### 3.2. Association between T3 and Cardiac Function

**Figure 2:**
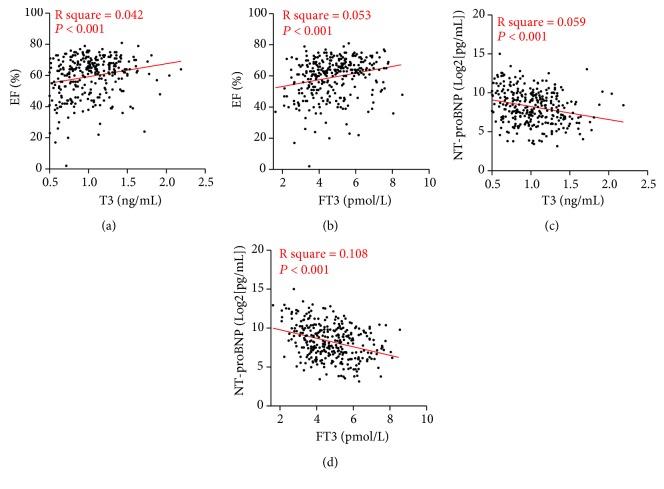
Linear analysis between heart function and triiodothyronine level. (a) Simple linear regression model with heart ejection fraction in relation to triiodothyronine level. (b) Simple linear regression model with heart ejection fraction in relation to free triiodothyronine level. (c) Simple linear regression model with NT-proBNP in relation to triiodothyronine level. (d) Simple linear regression model with NT-proBNP in relation to free triiodothyronine level.

As baseline characters exhibited a significant alteration of EF and NT-proBNP based on different T3 and FT3 levels, which were crucially important to evaluate cardiac function during AMI, we then carried on to investigate the relationship between T3 and cardiac function by regression analysis. The T3 levels were found to be significantly positively correlated with EF (*R* square = 0.042, *P* < 0.001) and negatively correlated with admission NT-proBNP levels (*R* square = 0.059, *P* < 0.001), which is the same with the correlation between FT3 and EF (*R* square = 0.053, *P* < 0.001) and admission NT-proBNP levels (*R* square = 0.108, *P* < 0.001) (Figure 2). Multiregression analysis was then utilized to further explain the association of T3 and cardiac function. Interestingly, although both T3 and FT3 levels were found to be significantly positively correlated with EF in AMI patients (*P* < 0.05), only FT3 was found to be significantly negatively correlated with NT-proBNP (*P* < 0.05). In addition, TSH, thyroid-stimulating hormone, was also found to be positively correlated with EF (*P* < 0.05) (Table 3). It is noteworthy that the TSH, T4, and FT4 did not exhibit a significant correlation with cardiac function in the present cohort.

### 3.3. All-Cause Mortality and MACE

**Figure 3:**
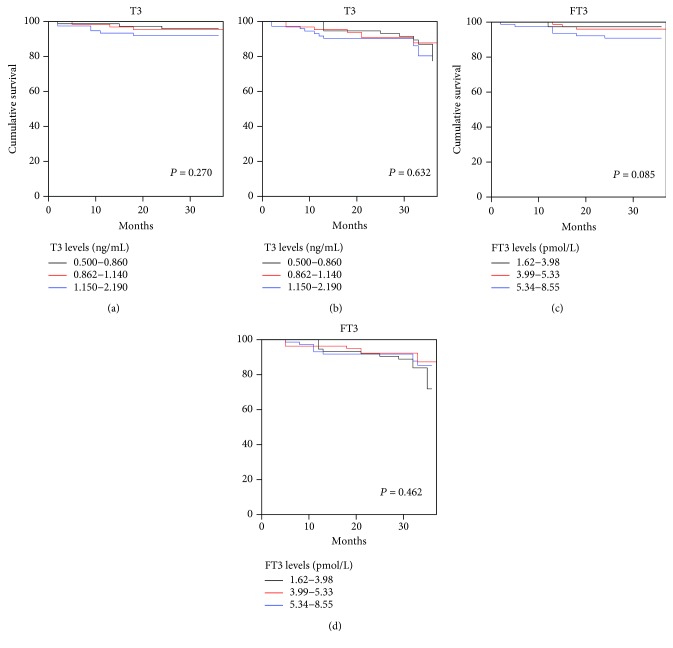
Kaplan-Meier survival curves for freedom from all-cause mortality and MACE judging by triiodothyronine level. (a) Kaplan-Meier survival curve for freedom from all-cause mortality judging by triiodothyronine level. *P* = 0.270. (b) Kaplan-Meier survival curves for freedom from MACE judging by triiodothyronine level. *P* = 0.632. (c) Kaplan-Meier survival curves for freedom from all-cause mortality judging by free triiodothyronine level. *P* = 0.085. (d) Kaplan-Meier survival curves for freedom from MACE judging by free triiodothyronine level. *P* = 0.462. There is no significantly higher event-free survival rate in high triiodothyronine and free triiodothyronine levels.

During the follow-up period, 12 (5.2%) patients died for all cause, 9 (4.0%) for cardiac cause, 28 (11.6%) for acute heart failure, and 12 (4.4%) had new-onset myocardial infarction. We also examined the prognostic significance of T3 and FT3 in AMI. As demonstrated in the Kaplan-Meier plots, the analysis revealed no significant difference with regard to different T3 or FT3 levels, as shown in Figure 3.

## 4. Discussion

In this study, serum FT3 level was found to be moderately associated with the cardiac function, as indicated by EF and NT-proBNP levels, on admission in patients with acute myocardial infarction. However, neither T3 nor FT3 exhibited effects on all-cause mortality rate and MACE in AMI patients during 2-year follow-up.

The important implication of the present study is that T3 levels are found to be correlated with the cardiac function as indicated by EF and NT-proBNP in AMI patients. In vitro studies have shown that T3 activates and regulates cardiac genes encoding proteins such as the myosin heavy chain isoforms and the sarcoplasmic reticulum calcium-activated ATPase pump [19–21], indicating a possible relationship between T3 level and cardiac function. Previous study has also indicated that T3 predicts outcomes in patients with acute heart failure [11]. In addition, low T3 is found out to be associated with elevation of N-terminal probrain natriuretic peptide (NT-proBNP) and mortality in dialysis patients [13, 22, 23]. However, few studies have addressed the question whether thyroid function is related to cardiac function during acute myocardial infarction. The major outcome of this study shows a positive correlation between T3 and FT3 levels and cardiac ejection fraction, indicating that T3 level is a potential therapeutic target for cardiac function improvement during acute myocardial infarction.

In the present analysis, it is noteworthy that the TSH, T4, and FT4 did not exhibit a significant correlation. The results correlate with previous publications that low T3 level has been proven to be correlated with myocardial damage in animal models. It has been known that T4 is solely a product of the thyroid gland, while T3 is a product of the thyroid and of many other tissues. Thus, the difference in the correlation results among thyroid hormones might reflect differential thyroid metabolism and function in AMI patients and requires further mechanism investigation.

Our study also shows that T3 and FT3 levels do not predict adverse cardiovascular outcomes in AMI patients. There is conflicting evidence regarding thyroid hormone levels and cardiovascular outcomes [24–26]. It has been reported that thyroid function tests predict disability in advanced old age patients, with higher serum TSH levels predicting better outcomes [27]. Moreover, in patients with acute heart failure, subclinical hypothyroidism on admission is an independent predictor of adverse cardiovascular outcomes [24]. In this study, thyroid function is not associated with all-cause mortality and MACE in AMI patients over a 2-year follow-up, indicating that the interaction between T3 and cardiac function might be more significant during acute phase of myocardial infarction. Meanwhile, it is also noteworthy that the less clear association between T3 and cardiovascular outcomes could be due to a limited number of patients with a relatively short follow-up in the present study, and its association with long-term prognosis may be stronger. As a result, more well-designed and long-term studies as well as systemic analysis are needed to investigate whether thyroid function will play an important role in the prognosis of AMI.

The present study also has several limitations. On the one hand, this is a single-centre-based observational cohort study. As the sample size in this study is relatively small, thus, the comparisons of subgroups may lack power to detect significant differences for selected variables, especially in Kaplan-Meier survival analysis. Notably, the association between T3 levels and cardiac function as interpreted by EF and NT-proBNP exhibits only low correlation coefficients, indicating at best a moderate association. On the other hand, the onset of AMI and the coronary intervention vary among patients. As a result, the correlation between AMI progression, iodine intake, and thyroid function could lead to a confounding bias and limit the interpretation of pathophysiology of thyroid function during the onset and progression of AMI. Therefore, multicentre-based clinical observations and subgroup analysis are necessary to further evaluate the predictive value of T3 for MACE and all-cause mortality, so as to explore the therapeutic value of modulating thyroid function during AMI.

In conclusion, in patients with AMI, T3 and FT3 levels are moderately positively correlated with cardiac function on admission in AMI patients but did not predict a long-time survival rate. The results of this study further provide therapeutic view that thyroid function control could be one of the treatment targets for AMI patients. Further studies are needed to determine whether longer-term follow-up would further identify the prognosis effect of T3 on MACE and all-cause mortality.

## Figures and Tables

**Table 1:** Basic characteristics based on T3 levels in AMI patients.

	Triiodothyronine levels (ng/mL)			*P* value
	0.500–0.860	0.862–1.140	1.150–2.190	
Female (%)	22.02%	24.77%	24.77%	
GRACE score	112.252 ± 42.084	127.159 ± 40.671	129.294 ± 38.054	<0.01
Age (y)	58.766 ± 10.313	59.477 ± 10.039	59.725 ± 10.237	0.860
HR (bpm)	70.355 ± 21.222	71.336 ± 16.876	70.706 ± 14.019	0.933
Onset of AMI (h)	20.077 ± 13.130	13.538 ± 16.033	18.385 ± 30.321	0.602
CKMB (U/L)	33.371 ± 58.571	48.333 ± 70.930	41.862 ± 60.303	0.210
HbA1C (%)	5.748 ± 0.340	5.744 ± 0.361	5.718 ± 0.345	0.453
sBP (mmHg)	124.542 ± 21.078	126.453 ± 16.723	125.303 ± 14.810	<0.05
dBP (mmHg)	79.075 ± 13.482	78.189 ± 10.272	78.083 ± 10.610	0.061
EF (%)	55.059 ± 14.298	61.697 ± 10.699	61.879 ± 11.132	<0.001
AST (U/L)	77.610 ± 79.248	44.818 ± 49.386	34.039 ± 32.576	<0.001
Creatine (*μ*mol/L)	69.688 ± 16.694	67.767 ± 15.181	69.090 ± 14.364	0.708
NT-proBNP (pg/mL)	1561.706 ± 3620.966	552.291 ± 758.825	570.607 ± 1117.933	<0.001
T4 (*μ*g/dL)	6.380 ± 1.666	7.151 ± 1.545	8.053 ± 1.808	<0.001
T3 (ng/mL)	0.700 ± 0.102	1.009 ± 0.078	1.377 ± 0.195	<0.001
FT4 (pmol/L)	13.738 ± 2.894	15.028 ± 2.991	16.350 ± 3.136	<0.001
FT3 (pmol/L)	4.079 ± 1.124	4.792 ± 1.149	5.628 ± 1.145	<0.001
TSH (*μ*IU/mL)	2.782 ± 7.471	1.960 ± 1.408	2.748 ± 3.519	0.315
TGAB (%)	9.932 ± 13.510	6.797 ± 9.021	7.567 ± 10.325	0.117
TMAB (%)	7.295 ± 8.741	5.323 ± 6.282	5.595 ± 6.718	0.119
Previous history of hypertension (%)	47.71%	54.13%	52.29%	
CHF (%)	10.09%	7.34%	5.50%	
Myocardial infarction (%)	17.43%	19.27%	16.51%	
PCI or CABG (%)	22.02%	22.02%	21.10%	
In-hospital treatment				
Aspirin (%)	95.41%	97.25%	97.25%	
*β*-Blocker (%)	81.65%	88.07%	79.82%	
Statin (%)	97.25%	99.08%	93.58%	
CCB (%)	19.27%	23.85%	17.43%	

Abbreviations: GRACE: the Global Registry of Acute Coronary Events; HR: heart rate; CKMB: MB isoenzyme of creatine kinase; HbA1c: hemoglobin A1c; BP: blood pressure; EF: ejection fraction; AST: aspartate transaminase; NT-proBNP: probrain natriuretic peptide; T4: thyroxine; T3: triiodothyronine; FT4: free thyroxine; FT3: free triiodothyronine; TSH: thyroid-stimulating hormone; TGAB: thyroglobulin; TMAB: thyroid microsomal antibody; CHF: chronic heart failure; PCI: percutaneous coronary intervention; CABG: coronary artery bypass graft; CCB: calcium channel blocker. Data are mean ± SD and number (%).

**Table 2:** Basic characteristics based on FT3 levels in AMI patients.

	Free triiodothyronine levels (pmol/L)			*P* value
	1.62–3.98	3.99–5.33	5.34–8.55	
Female (%)	18.35%	18.35%	22.94%	
GRACE score	116.098 ± 43.037	121.991 ± 40.497	128.117 ± 38.421	0.091
Age (y)	59.625 ± 10.468	58.351 ± 10.107	59.694 ± 10.490	0.550
HR (bpm)	69.732 ± 14.248	72.728 ± 22.362	69.856 ± 13.955	0.344
Onset of AMI (h)	17.538 ± 32.545	17.769 ± 30.339	16.692 ± 18.481	0.911
CKMB (U/L)	40.333 ± 66.052	40.266 ± 61.909	41.398 ± 60.269	0.989
HbA1C (%)	124.027 ± 19.070	124.513 ± 18.211	125.973 ± 16.001	0.272
sBP (mmHg)	76.929 ± 11.943	78.796 ± 11.498	78.369 ± 11.353	0.700
dBP (mmHg)	5.693 ± 0.356	5.744 ± 0.357	5.767 ± 0.328	0.454
EF (%)	67.939 ± 14.945	69.152 ± 16.936	69.203 ± 14.298	<0.001
AST (U/L)	55.486 ± 13.468	60.057 ± 11.187	62.135 ± 11.974	<0.05
Creatine (*μ*mol/L)	68.437 ± 75.677	53.158 ± 57.580	40.438 ± 45.711	0.192
NT-proBNP (pg/mL)	1780.395 ± 3700.982	739.469 ± 1208.398	411.908 ± 537.265	<0.001
T4 (*μ*g/dL)	6.464 ± 1.641	7.005 ± 1.687	7.911 ± 1.875	<0.001
T3 (ng/mL)	0.845 ± 0.238	1.015 ± 0.277	1.218 ± 0.286	<0.001
FT4 (pmol/L)	13.432 ± 2.918	14.832 ± 2.836	16.540 ± 3.075	<0.001
FT3 (pmol/L)	3.336 ± 0.512	4.690 ± 0.394	6.295 ± 0.750	<0.001
TSH (*μ*IU/mL)	2.812 ± 7.305	2.238 ± 3.348	2.241 ± 1.618	0.585
TGAB (%)	8.873 ± 12.438	6.765 ± 9.865	8.271 ± 10.397	0.335
TMAB (%)	6.567 ± 8.205	5.184 ± 6.313	6.202 ± 7.025	335.000
Previous history of hypertension (%)	44.95%	50.46%	54.13%	
CHF (%)	6.42%	1.83%	1.83%	
Myocardial infarction (%) PCI or CABG (%)	13.76%	13.76%	14.68%	
In-hospital treatment	18.35%	16.51%	20.18%	
Aspirin (%)	95.41%	97.25%	97.25%	
*β*-Blocker (%)	78.90%	88.99%	80.73%	
Statin (%)	97.25%	99.08%	92.66%	
CCB (%)	15.60%	19.27%	15.60%	

Abbreviations: GRACE: the Global Registry of Acute Coronary Events; HR: heart rate; CKMB: MB isoenzyme of creatine kinase; HbA1c: hemoglobin A1c; BP: blood pressure; EF: ejection fraction; AST: aspartate transaminase; NT-proBNP: probrain natriuretic peptide; T4: thyroxine; T3: triiodothyronine; FT4: free thyroxine; FT3: free triiodothyronine; TSH: thyroid-stimulating hormone; TGAB: thyroglobulin; TMAB: thyroid microsomal antibody; CHF: chronic heart failure; PCI: percutaneous coronary intervention; CABG: coronary artery bypass graft; CCB: calcium channel blocker. Data are mean ± SD and number (%).

**Table 3:** Multiregression analysis of NT-proBNP and EF.

Factors	Coefficient	SEM	*P* value
Multiregression analysis of NT-proBNP			
Age (y)	−0.001	14.246	0.994
HR (bpm)	0.161	7.271	<0.05
GRACE score	0.106	3.986	0.141
T3 (ng/mL)	−0.094	486.295	0.155
FT3 (pmol/L)	−0.195	115.569	<0.05
TSH (*μ*IU/mL)	−0.024	25.870	0.666
CKMB (U/L)	−0.045	2.294	0.480
Creatine (*μ*mol/L)	0.014	8.706	0.807
HbA1C (%)	−0.039	377.367	0.495
Multiregression analysis of EF			
Age (y)	−0.051	0.081	0.432
HR (bpm)	0.087	0.041	0.134
GRACE score	0.017	0.023	0.816
T3 (ng/mL)	0.132	2.687	<0.05
FT3 (pmol/L)	0.152	0.633	<0.05
TSH (*μ*IU/mL)	0.121	0.145	<0.05
CKMB (U/L)	0.021	0.013	0.744
Creatine (*μ*mol/L)	−0.117	0.052	0.054
HbA1C (%)	0.034	2.126	0.564

Abbreviations: HR: heart rate; GRACE: the Global Registry of Acute Coronary Events; T3: triiodothyronine; FT3: free triiodothyronine; TSH: thyroid-stimulating hormone; CKMB: MB isoenzyme of creatine kinase; HbA1c: hemoglobin A1c.

## Data Availability

The datasets analysed during the current study are available from the corresponding author on reasonable request.

## Abbreviations

AMI: Acute myocardial infarction
T3: Triiodothyronine
FT3: Free triiodothyronine
T4: Thyroxine
FT4: Free thyroxine
TSH: Thyroid-stimulating hormone
TGAB: Thyroglobulin
TMAB: Thyroid microsomal antibody
EF: Cardiac ejection fraction
NT-proBNP: N-terminal probrain natriuretic peptide
MACE: Major adverse cardiovascular events
GRACE: The Global Registry of Acute Coronary Events
HR: Heart rate
CKMB: MB isoenzyme of creatine kinase
HbA1c: Hemoglobin A1c
BP: Blood pressure
AST: Aspartate transaminase
CHF: Chronic heart failure
PCI: Percutaneous coronary intervention
CABG: Coronary artery bypass graft
CCB: Calcium channel blocker.
